# Role of Vitamin D Deficiency in Extraskeletal Complications: Predictor of Health Outcome or Marker of Health Status?

**DOI:** 10.1155/2015/563403

**Published:** 2015-05-05

**Authors:** Idris Guessous

**Affiliations:** ^1^Unit of Population Epidemiology, Department of Community Medicine and Primary Care and Emergency Medicine, Geneva University Hospitals, 1214 Geneva, Switzerland; ^2^Institute of Social and Preventive Medicine (IUMSP), Lausanne University Hospital, 1010 Lausanne, Switzerland; ^3^Department of Epidemiology, Rollins School of Public Health, Emory University, Atlanta, GA 1518, USA

## Abstract

The relationship of vitamin D with extraskeletal complications, such as cardiovascular disease, cancer, and autoimmune disease, is of major interest considering its roles in key biological processes and the worldwide high prevalence of vitamin D deficiency. However, the causal relationships between vitamin D and most extraskeletal complications are weak. Currently, a heated debate over vitamin D is being conducted according to two hypotheses. In this review, we first present the different arguments that suggest a major role of vitamin D in a very broad type of extraskeletal complications (hypothesis #1). We then present results from recent meta-analyses of randomized controlled trials indicating a lack of association of vitamin D with major extraskeletal complications (hypothesis #2). We discuss different issues (e.g., causality, confounding, reverse causation, misclassification, and Mendelian randomization) that contribute to the favoring of one hypothesis over the other. While ultimately only one hypothesis is correct, we anticipate that the results from the ongoing randomized controlled trials will be unlikely to reconcile the divided experts.

## 1. Introduction

In humans, most circulating vitamin D is synthesized from cholesterol following exposure to ultraviolet B (UVB) in sunlight, whereas a smaller amount is derived from diet and dietary supplements. Diet contributes only between 10% and 20% to 25(OH)D levels but becomes more important when sunshine exposure is low [1]. Fish is the major dietary source of vitamin D in humans.

**Figure 1:**
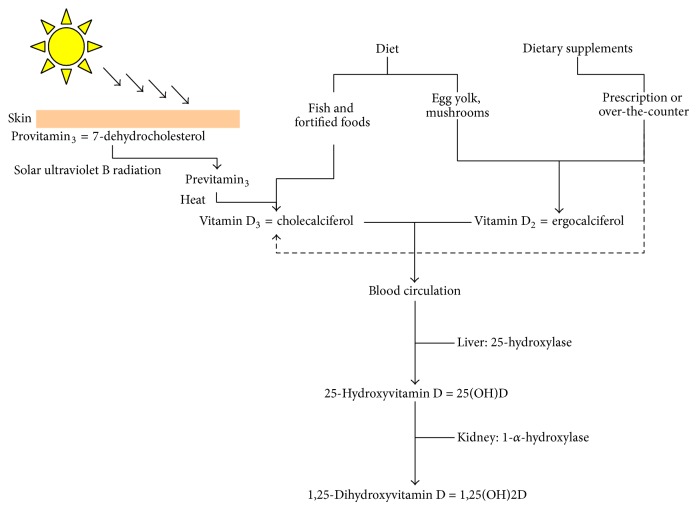
Sources of vitamin D and the first steps of vitamin D synthesis.

A summary of the synthesis and metabolism of vitamin D, which have been described in detail in multiple reviews and textbooks [1–6], is presented below. Calcitriol, or 1,25-dihydroxyvitamin D [1,25(OH)2D], is the hormonally active form of vitamin D that is derived from the following three sources: sunlight, diet, and dietary supplements (Figure 1).

There are two precursors to active vitamin D hormones, vitamins D_3_ (cholecalciferol) and D_2_ (ergocalciferol). Vitamin D_3_ is synthesized in the skin after exposure to UVB light. Solar UVB radiation (wavelength of 290 to 315 nm) penetrates the skin and converts 7-dehydrocholesterol to previtamin D_3_ by photolysis, which is rapidly converted to vitamin D_3_ [4]. Vitamin D_3_ may also be obtained from some dietary sources and dietary supplements. Vitamin D_2_ (ergocalciferol) is derived solely from the diet (and not from UVB). Both vitamin D_3_ and D_2_ enter the blood circulation and are attracted to the vitamin D binding protein (VDBP).

Vitamin D in the circulation is transported to the liver, which is where it is converted by vitamin D-25-hydroxylase to 25-hydroxyvitamin D [25(OH)D]. This first hydroxylation is catalyzed by the CYP27A1 enzyme. This form of vitamin D is thought to be biologically inactive and must be converted in the kidneys by 25(OH)D 1*α*-hydroxylase to its biologically active form, 1,25(OH)2D [2]. This second hydroxylation is catalyzed by CYP27B1, which is located in the inner mitochondrial membrane of the proximal tubule cells of the kidneys. The 24-hydroxylation of both 25(OH)D and 1,25(OH)D to form 24,25(OH)D and 1,24,25(OH)D is the primary mechanism and the first step towards the inactivation of vitamin D metabolites.

*Genomic and Nongenomic Vitamin D Functions*. The actions of vitamin D are largely mediated by genomic functions. Vitamin D interacts with nuclear vitamin D receptor (VDR). VDR is a ligand-induced nuclear receptor that regulates the expression of over 900 genes throughout the genome [7, 8]. It influences the transcription of genes that are responsive to the VDR-vitamin D complex. 1,25(OH)2D dissociates from serum VDBP and enters the cell. Inside the cell, it binds to and activates VDR, and the VDR-vitamin D complex translocates from the cytosol to the nucleus, where it is joined by the retinoid X receptor (RXR) partner [6]. The 1,25(OH)2D-VDR-RXR complex binds to specific sequences in the promoter regions of target genes that are called vitamin D response elements (VDREs), leading to the promotion and modulation of the expression of the targeted genes. 25(OH)D is less active than 1,25(OH)D2 because of its lower affinity for VDR.

Vitamin D has also some nongenomic rapid-response functions. In terms of its nongenomic functions, it functions as a steroid hormone by activating signal transduction pathways linked to vitamin D receptors on cell membranes.

## 2. Hypothesis #1: Vitamin D Is Causally Associated with Extraskeletal Complications

### 2.1. Vitamin D Deficiency and Associated Seasonal and Geographic Patterns

#### 2.1.1. High Prevalence of Vitamin D Deficiency

The worldwide high prevalence of vitamin D deficiency is often presented as being clearly linked with the high burden of extraskeletal complications. In Europe, the mean serum 25(OH)D levels (conversion factor for 25(OH)D: 1 ng/mL = 2.496 nmol/L) reported in population-based studies have varied from 18 ng/mL (29 nmol/L) in Italy to 30 ng/mL (75 nmol/L) in Norway but are generally very low [9]. A recent review of vitamin D deficiency in central Europe has concluded that 25(OH)D levels are on average below 30 ng/mL [10]. Worldwide, it is estimated that one billion people have vitamin D deficiency [11], and it affects more than 40% of US and European men and women [12]. This high prevalence has also been reported in a Swiss study that has calculated a population-based estimation of vitamin D deficiency according to 25(OH)D serum levels, reporting that 39.5% of the population has 25(OH)D levels of <20 ng/mL (<50 nmol/L) (vitamin D deficiency) [13].

Of course, the prevalence of vitamin D deficiency and insufficiency depends on the definition used. Several definitions of vitamin D deficiency exist, some of which are presented in Table 1. The reasons for the differences in these definitions are partially due to the outcomes, for which the defined cutoff levels were different (e.g., bone fracture, parathyroid hormone (PTH) level, cardiovascular (CV) events, and cancer). Obviously, it is very unlikely that the risks of these outcomes all increase at the same 25(OH)D level. Given the wide variation in the definitions of vitamin D deficiency, Pilz et al. have suggested that the ideal 25(OH)D concentration for overall health-related outcomes ranges from 40 nmol/L to 120 nmol/L [14]. In fact, a more accurate range seems to be between 40 and 80 nmol/L according to a recent study that has suggested the presence of a J-curve between vitamin D level and overall mortality [15]. Debates on the ideal vitamin D concentration have increased after the publishing of the 2011 Institute of Medicine (IOM) report [16]. The IOM suggested that a lower 25(OH)D level should be used to define vitamin D deficiency and that a 25(OH)D serum level of 20 ng/mL (50 nmol/L) is desirable for bone and overall health [16]. While vitamin D deficiency defined as a serum 25(OH)D level of <20 ng/mL (50 nmol/L) and vitamin D insufficiency defined as a 25(OH)D level of between 20 and 29 ng/mL (50–75 nmol/L) have been extensively used in epidemiological studies, the definition of vitamin D insufficiency is more controversial. Of note, a 25(OH)D level of at least 20 ng/mL (50 nmol/L) was chosen by the IOM, with recommended dietary allowances of 600 IU/day of vitamin D for individuals aged 1–70 years and 800 IU/day for those aged 71 years and older to meet the requirements of at least 97.5% of the population. Yet other definitions of vitamin D deficiency and insufficiency are proposed in the Kidney Disease Improving Global Outcomes (KDIGO) guidelines [17]; serum levels of vitamin D are considered as adequate when the concentration of 25(OH)D is higher than 30 ng/mL, levels between 15–30 ng/mL are considered as insufficient, and values that are lower than 15 ng/mL define the diagnosis of vitamin D deficiency (Table 1).

Several factors have been proposed to explain the high prevalence of vitamin D deficiency, one of which is inadequate vitamin D intake [18]. Using data from a 10-year trend study (1999–2009, *N* = 9,320) performed in Geneva, Switzerland, de Abreu et al. have found that slightly more than 10% of participants complied with dietary recommendations for vitamin D intake [19]. This finding was in line with data suggesting that vitamin D is one of the critical vitamins and that its intake is below the recommended level [18, 20]. This chronic insufficient intake of micronutrients in a population without the emergence of immediate clinical signs is typical of “Hidden Hunger” [20, 21].

#### 2.1.2. Latitude and Season

Vitamin D is often presented as the link explaining most of the observed correlations between latitude and season with extraskeletal complications. Most vitamin D is produced in the skin from 7-dehydrocholesterol by sunlight UVB exposure, which varies with latitude and season [3]. UVB exposure decreases from the equator towards the polar regions [22]. At approximately 0° latitude (i.e., the equator, e.g., the Republic of Seychelles), a high level of vitamin D-effective UV radiation is present, which varies only slightly throughout the year. On the other hand, at approximately 40° latitude (e.g., Switzerland, with a latitude of 47°), the level of vitamin D-effective UV radiation varies greatly throughout the year and decreases substantially during the winter [23].

Ecological studies have reported an inverse correlation of ischemic heart disease with sunlight and a seasonal pattern of coronary heart disease mortality [24]. We contributed to the largest (*N* = 230,000 from 15 countries) and most comprehensive (body mass index, waist circumference, blood pressure, total high- (HDL) and low-density lipoprotein (LDL) cholesterol, triglycerides, and glucose levels) study ever conducted to assess the seasonality of cardiovascular risk factors [25]. The results have strongly suggested that cardiovascular risk factors present a seasonal pattern, with lower levels occurring during the summer and higher levels during the winter, suggesting that at least part of the patterning might be due to changes in air/outdoor temperature, air pollution, and exposure to sunlight and thus differences in vitamin D levels [25].

Geographic and seasonal patterns of cancer incidence and mortality have also been reported in ecological studies [26, 27], suggesting a correlation between sunlight exposure (and, thus, vitamin D) and cancer.

### 2.2. VDR Receptor,* VDR* Gene, and Extrarenal 1-Alpha-Hydroxylase

The wide distribution of VDRs in humans, the influence of vitamin D on more than 3% of the human genome, and the extrarenal presence of 1-alpha-hydroxylase are often presented as factors explaining the very broad influence of vitamin D on health and disease [20].

Several biological systems have VDRs and are responsive to vitamin D. Because one of the major biological functions of this vitamin is to maintain calcium homeostasis, typical responses occur in the intestines and kidneys, which is where 1,25(OH)2D-VDR regulates genes, leading to increased calcium and phosphate absorption. Another typical action of vitamin D is the suppression of PTH synthesis by 1,25(OH)2D-VDR in the parathyroid glands. However, a recent study has reported that the 1,25(OH)2D-VDR complex controls the expression of genes and syntheses of mRNAs that are unrelated to calcium homeostasis. In fact, one of the reasons for the recent growing interest in the role of vitamin D in extraskeletal complications is emerging evidence that VDRs are largely distributed throughout human tissues. The list of tissues in which VDRs are distributed includes the hepatogastrointestinal system (e.g., the colon), the respiratory system (e.g., the lungs), the central nervous system (e.g., neurons), the cardiovascular system (e.g., cardiomyocytes), and the kidneys [28]. Thus, tissue and cellular VDR distribution is wide. Most human tissues and cell types are responsive to 1,25(OH)2D [29, 30]. For example, the vitamin D-VDR complex controls the expression of genes involved in the inhibition of the renin-angiotensin system (RAS), which influences blood pressure, as well as genes that promote the secretion of insulin and cell proliferation and differentiation [31–34].

VDR abundance and activity seem to play important roles in individual responses to 1,25(OH)2D. Some VDR abundance and activity are determined by* VDR* polymorphisms (i.e., genetic variations that occur at a frequency of >1% [35]) [5]. The* VDR* gene lies on chromosome 12 [36]. The coding sequence of the VDR protein is comprised of eight exons, and several (>60) genetic polymorphisms have been identified [5, 37]. These polymorphisms may alter transcriptional activity and thus VDR abundance and may modulate cellular responsiveness to 1,25(OH)2D. In particular, there is a polymorphic site at the 5′ end of the* VDR* gene* Fok I* C/T. This polymorphism, in contrast with the other* VDR* variants, results in an altered amino acid sequence [5, 37]. The* Fok I* C allele generates a shorter VDR protein than the T allele, and this shorter protein is thought to be more active than the longer one [5]. Another important polymorphism is the* Cdx2 VDR* polymorphism, which is located in the promoter region of the* VDR* gene in exon 1. The* Cdx2 VDR* polymorphism has been associated with VDR transcriptional activity in the intestinal tract. The T allele has shown up to 70% greater transcriptional activity compared with the C allele [38].

It was first estimated that the number of primary 1,25(OH)2D target genes is in the order of 100–500 per tissue, but there seems to be many more genomic VDR binding sites per cell type (between 1000 and 10,000) [29]. However, as stressed by Carlberg, some of these sites may not have specific functions and may only represent noise [29].

The observations that extrarenal 1,25(OH)2D can also be produced (extrarenal 1-alpha-hydroxylase) and that 1,25(OH)2D can act locally in the tissues where it is produced (autocrine/paracrine activities) also support the potential role of vitamin D in extraskeletal complications [39].

### 2.3. Evidence from Molecular and Animal Studies

Molecular and animal studies have established associations of vitamin D with extraskeletal complications. Given the abundance of available literature on this topic, we will briefly present some major mechanisms involved in specific but frequent chronic extraskeletal complications, namely, CV disease (CVD) (high blood pressure, coronary heart disease, and stroke), type II diabetes, cancer, and chronic kidney disease (CKD).

#### 2.3.1. Cardiovascular Disease: High Blood Pressure and Coronary Heart Disease

Molecular evidence has revealed effects of 1,25(OH)2D on blood pressure-related mechanisms [34]. These mechanisms include the direct inhibition by 1,25(OH)2D of the RAS. VDR is expressed in the juxtaglomerular apparatus and modulates renin synthesis. Mice lacking VDRs are hyperreninemic and present with high blood pressure and cardiac hypertrophy [40]. By contrast, the overexpression of VDR in the mouse juxtaglomerular apparatus leads to hyporeninemia [41]. In addition, vitamin D can regulate blood pressure through the prevention of secondary hyperparathyroidism [42], and it seems to have a direct effect on vascular cells and endothelial function [43].

Different mechanisms have been proposed to explain the association of vitamin D with coronary heart disease [44], some of which are indirect. Vitamin D could be related to coronary heart disease by affecting blood pressure, glycemic control, or PTH. Other proposed mechanisms are more directly related to atherosclerosis, cardiac tissues, and vasculature. Animal studies have highlighted the roles of vitamin D in cardiomyocyte remodeling in response to injury and atherosclerosis as well as in cardiac relaxation and contractility [45]. Serum levels of vitamin D seem to be inversely associated with the extent of vascular calcification in individuals at risk of ischemic heart disease [44, 46]. Vitamin D could confer protection against atherosclerosis and vascular calcification by directly affecting vascular smooth muscle cells (VSMCs). This vitamin causes an acute influx of calcium in VSMCs that might inhibit their proliferation [47]. Vitamin D could also be associated with coronary heart disease by downregulating proinflammatory cytokines (e.g., TNF-*α* and IL-6) and upregulating the anti-inflammatory cytokine IL-10 [44].

#### 2.3.2. Type II Diabetes and Cancer

There is great interest in the roles of vitamin D in the susceptibilities of type II diabetes and metabolic syndrome [47]. The effects of vitamin D on type II diabetes could be mediated by its role in pancreatic *β*-cell function, insulin resistance, or inflammation [33, 48–50]. With respect to cancer, studies using animal models have shown that knocking out* VDR* induces many types of cancers, including mammary, prostate, and colon cancers [26, 51]. Recently, a low vitamin D level has even been proposed as a modulator of the link between diabetes and cancer [52].

#### 2.3.3. Chronic Kidney Disease

**Figure 2:**
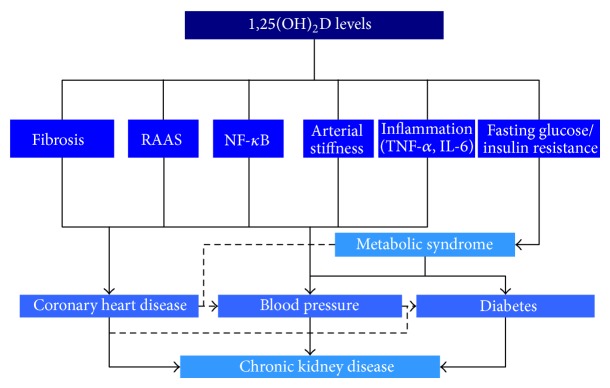
Main hypothetical mechanisms of action of vitamin D in chronic kidney disease. Mechanisms related to parathormone and calcium are not displayed. VSMC = vascular smooth muscle cells; RAAS = renin-angiotensin-aldosterone system; NF-*κ*B = nuclear factor-*κ*B; TNF-*α* = tumor necrosis factor-*α*; IL-6 = interleukin-6.

CKD is defined as the persistence for 3 or more months of structural and/or functional abnormalities of the kidney [53]. CKD is associated with an increased risk of ischemic heart disease, stroke, peripheral vascular disease, anemia, bone disease, end-stage renal disease, and mortality. Mechanisms (beyond those related to parathormone or calcium) by which vitamin D might be associated with CKD are summarized in Figure 2. Vitamin D insufficiency correlates with mortality risk among patients with CKD and evidence has been reviewed elsewhere [54, 55]. For example, a recent meta-analysis of 20 observational studies showed vitamin D treatment to be associated with decreased risk of all-cause and cardiovascular mortality in patients with CKD not requiring dialysis and patients with end stage renal disease (ESRD) requiring dialysis [55]. This meta-analysis also highlighted the need of well-designed randomized controlled trials to formally assess the survival benefits of vitamin D.

One of the mechanisms associating vitamin D with CKD involves the nuclear factor-*κ*B (NF-*κ*B) pathway. NF-*κ*B is a family of transcription factors that functions as a master regulator of the immune response [56]. It regulates a wide range of genes involved in inflammation, proliferation, and fibrogenesis and is known to have a key role in kidney disease [57]. A direct inhibition by 1,25(OH)2D of the (NF-*κ*B) pathway has been reported. Both RAS and NF-*κ*B promote the production of profibrotic and proinflammatory factors, increase oxidative stress, and damage podocytes.

### 2.4. Evidence from Observational Studies

Guessous et al. have previously reviewed evidence of the roles of vitamin D in hypertension, coronary heart disease, and stroke [34]. Out of four observational prospective studies identified, two have reported an inverse association between 25(OH)D level and hypertension risk, one found no such association, and one found an association neither with hypertension nor with changes in blood pressure. Lower vitamin D levels have been found to be associated with an increased risk of myocardial infarction in a prospective study of men aged 40–75 years [58]. Higher levels of 1,25(OH)D have been associated with decreased risk of stroke among participants in a cohort study conducted in Finland [59].

### 2.5. Economic Impact of Correcting Vitamin D Deficiency

Finally, a potentially huge economic benefit resulting from the correction (at the population level) of vitamin D deficiency is often presented. Assuming that vitamin D deficiency is causally associated with extraskeletal complications, it has been suggested that a rise in the serum 25(OH)D levels of all Europeans to 40 nmol/L (achieved by a daily intake of 2000–3000 IU of vitamin D) would lower health-care costs by up to 17%, which represents a reduction of 187,000 million Euros/year [20, 60]. Thus, a single, simple intervention could have a major impact on the economy of health care. However, this belief is conditional on a causal relationship of vitamin D with extraskeletal complications that is far from being accepted as described below.

## 3. Hypothesis #2: Vitamin D Is Not Causally Associated with Extraskeletal Complications

The belief that vitamin D is not causally associated with extraskeletal complications is mainly based on the absence of robust evidence of this association from randomized controlled trials (RCTs), on the potentially unfavorable effects of vitamin D supplementation and on the precedents of other vitamin and antioxidant trials.

### 3.1. Randomized Controlled Trials

To date, most associations of vitamin D with extraskeletal complications reported by nonexperimental studies have not been replicated in RCTs. For example, results from observational cross-sectional and prospective studies on the inverse association of vitamin D intake or 25(OH)D level with blood pressure have not been confirmed in RCTs [34]. Only one out of three RCTs identified have found a decrease in blood pressure in the intervention arm, but daily supplementation included both vitamin D_3_ and calcium (not vitamin D only) [61]. Two RCTs primarily designed to determine the effect of vitamin D supplementation on the risk of fracture have reported conflicting effects of this vitamin on coronary heart disease [62, 63], and no RCT has evaluated the effect of vitamin D supplementation on coronary heart disease as a primary outcome. Trivedi et al. have found no effect of vitamin D supplementation on stroke [62].

To evaluate the presence of biases in the associations of vitamin D with diverse skeletal and extraskeletal outcomes, Theodoratou et al. have performed a review of evidence obtained from systematic reviews and observational meta-analyses and RCTs (i.e., an umbrella review) [64]. More than 260 systematic reviews or meta-analyses that have included over 130 outcomes have been examined. This group has reported a lack of highly convincing evidence of a clear link of vitamin D with any outcome. Even for skeletal complications, the authors have concluded that RCTs that examined vitamin D only (without calcium supplementation) have failed to demonstrate protective effects of vitamin D supplementation on fractures or falls. Moreover, a 2014 trial sequential meta-analysis (i.e., analysis that modeled the changing precision in estimates of effects as trials are reported and the likely effects of future trial results on the existing body of data) has shown that the effects of vitamin D supplementation on extraskeletal complications are below the futility boundary of 15% [65, 66]. Thus, future trials are unlikely to alter the conclusion of no causal association.

A 2009 narrative review has estimated that raising the minimum year-round serum 25(OH)D level to 100–150 nmol/L (40 to 60 ng/mL) would prevent approximately 58,000 new cases of breast cancer and 49,000 new cases of colorectal cancer each year in the US and Canada [67]. However, the most recent (2011) meta-analysis has concluded that because of the potential confounding data inherent in observational studies and the limited data obtained from RCTs, evidence is currently insufficient to draw conclusions about the efficacy of vitamin D supplementation for cancer prevention [68]. A 2014 review of vitamin D status and CVD by the UK Nutrition Society has concluded that data supporting a causal link between vitamin D status and CVD are mixed and ambiguous [69]. Similar conclusions were independently published in 2011 by the North American DRI committee [16], by the Endocrine Task Force [70], and more recently, by others [65, 71–74]. In terms of endocrine health and disease, available evidence does not show that vitamin D supplementation consistently decreases the risk of type II diabetes, Addison's disease, or autoimmune thyroid disease [75].

In a 2014 meta-analysis of observational studies (*N* = 73) and RCTs (*N* = 22) of vitamin D and mortality caused by extraskeletal complications, Chowdhury et al. have found evidence that 25(OH)D level is inversely associated with risk of death due to extraskeletal complications, but no evidence of such a relationship has been found in RCTs [76]. Stratified analysis has suggested that supplementation with vitamin D_3_ and not D_2_ reduces all-cause mortality [76], but most of the trials included in the stratified analysis reported a high score for the risk of bias.

Overall, the conclusions of the meta-analyses and reports are in contrast with other recent reviews, which have concluded that adequate vitamin D supplementation is an important prophylactic factor for immunity, autoimmunity, CVD, cancer, fertility, pregnancy, dementia, and mortality [77]. Therefore, most scientists have acknowledged that the prevalence of vitamin D deficiency is high (although as discussed above, the definition of vitamin D normality is not universal); however, based on the lack of robust evidence, some have questioned whether it is a health problem [78].

### 3.2. Unfavorable Effects of Vitamin D

Vitamin D may have unfavorable effects. For example, it could potentially contribute to arterial stiffening and hypertension. Richart et al. [79] have proposed mechanisms of the renal versus extrarenal activation of vitamin D in relation to atherosclerosis, arterial stiffening, and hypertension. Macrophages in atherosclerotic lesions can locally activate 25(OH)D to form calcitriol, which could act as a vasoactive and prooxidative substance in VSMCs. Another concern is related to severe cases of hypercalcemia observed in children with* CYP24A1* mutations [80]. Concerns extend beyond extraskeletal complications because recent evidence has also suggested that high doses of vitamin D may increase the risks of fractures and falls [81, 82].

### 3.3. Vitamins, Antioxidants, and Hormone Replacement Therapy Precedents

Skepticism about the curative and/or predictive potentials of vitamin D for extraskeletal complications has also been magnified by previous vitamin-related research, in which evidence reported in observational studies has not been replicated in RCTs [83]. RCTs of beta-carotene, vitamin A, and vitamin E have not replicated findings from observational studies [84]. Even worse, RCTs have reported that some antioxidants and vitamins with supposed favorable effects (based on observational studies) in fact have unfavorable effects (based on RCTs). In 2008, the SELECT trial of vitamin E and selenium in cancer prevention was stopped prematurely because the intervention arm was more (although not statistically significantly) likely to develop prostate cancer [85]. This, of course, echoes the experience and lessons learned from the Women's Health Initiative (WHI) study on hormone replacement therapy (HRT) [86]. This trial was stopped prematurely when an increase in invasive breast cancer achieved statistical significance. This trial also found increases in heart attacks and strokes in the intervention group. These findings contrasted dramatically with conclusions of previous epidemiologic studies, which indicated a lack of a convincing link between breast cancer and HRT and reported that HRT decreases cardiovascular events [87].

## 4. Issues Favoring One Hypothesis over the Other

### 4.1. Causality

A major point in the debate about the real impact of vitamin D deficiency on extraskeletal complications is whether 25(OH)D belongs to the causal pathway. In other words, does a low 25(OH)D level cause disease or is it simply a side effect of either the exposure (e.g., sedentary behavior or obesity) or disease (e.g., cancer or autoimmune disease)? While an RCT (i.e., the supreme paradigm for epidemiological research [88]) is the best study design to determine causality, risk factors, such as low 25(OH)D levels, cannot be assessed with this type of trial for obvious ethical reasons. Observational studies, which are prone to spurious results, are then conducted. This issue of study design is so central that some epidemiologists have categorized the field into* descriptive* problems (i.e., a parameter of occurrence is related to a determinant without a causal interpretation of the relationship) and* causal* problems [88]. A causal interpretation of the relationship of an outcome parameter (type II diabetes) to a determinant (25(OH)D) can be given as long as the relationship is conditional on the entire set of potential confounders (both known and unknown). This requisite conditionality is best pursued by randomization [88] and thus the use of RCTs.

### 4.2. Confounding and Reverse Causation

Confounding happens when the effect of at least one extraneous factor (e.g., body mass index, BMI) is mixed with the effect of an exposure of interest (25(OH)D), thus distorting the estimate of the latter [89]. With respect to vitamin D, the list of potential known confounders is very large and includes the following major ones. (1) Age: the production of vitamin D by the skin decreases with age [2]. (2) BMI: the reasons for the inverse association between BMI and 25(OH)D level are not completely understood, but fat in the skin seems to decrease the efficacy of vitamin D synthesis, which may be due to 7-dehydrocholesterol sequestration. Of note, this inverse association could also be confounded by a decrease in sun exposure (e.g., a decrease in outdoor physical activity or comorbidities). (3) Latitude and seasons: theoretically, persons living in regions closer to the equator should present with higher levels of vitamin D synthesis than those residing in regions remote from the equator. In practice, however, because more than 90% of vitamin D arises from sunlight (in the absence of supplementation), levels of this vitamin also depend on cultural behaviors (clothing, time spent outdoors, and sunbathing habits). Overall, reports of the effect of latitude on serum 25(OH)D have been inconsistent [90–92]. A positive correlation between 25(OH)D and latitude has been found in a (25 European countries) pooled analysis, whereas the highest rate of 25(OH)D deficiency has been observed in Scottish participants (highest latitude) in a British cross-sectional study [91]. However, recent metaregression analysis did not find an influence of latitude on 25(OH)D level [92]. (4) Skin pigmentation: the packaging and sizes of melanosomes in keratocytes influence the darkness of the skin. Dark pigments in the skin reduce its ability to synthesize vitamin D from sunlight by up to 95% [22]. This fact likely explains why African-Americans have lower 25(OH)D than non-Hispanic whites in the US [22]. The darker skin pigmentation in individuals residing in southern compared to northern European countries may underlie the higher prevalence of 25(OH)D deficiency in southern Europe [93]. (5) Diet: diets typically contain only small amounts of vitamin D (vitamin D_3_ or vitamin D_2_). Fish is the major dietary source of vitamin D in humans. Three ounces of cooked salmon and 3.5 ounces of cooked mackerel provide 90% and 86%, respectively, of the recommended daily vitamin D intake (400–600 international units/day), whereas 3.5 ounces of cooked beef only provides 4% of the recommended intake. (6) Occasional sunscreen: sunscreen use by children and young adults is unlikely to cause vitamin D deficiency, but the chronic use of sunscreen by elderly individuals has been shown to decrease 25(OH)D and to cause vitamin D deficiency. (7) Altitude, air pollution, ozone, time of the day, and cloud cover: at higher altitudes, UVB radiation is stronger because the concentrations of aerosols and particles are lower. Air pollution decreases vitamin D-effective radiation. Ozone, time of the day, and cloud cover also influence vitamin D-effective radiation and thus vitamin D photosynthesis. Other potential confounders include genetic and epigenetic (i.e., heritable and modifiable changes in gene expression that do not affect DNA sequences) variations that have been implicated in association with vitamin D that may contribute to the interindividual variability of the impacts of its deficiency and/or supplementation [29, 94]. Disregarding the unknown potential confounders, fully accounting for all of these potential known confounders is practically impossible (residual confounding) unless randomization is used.

Temporality in causal criteria refers to the necessity that the cause precedes the effect in time (causal sequence) [89]. Thus, reverse causation refers to a situation in which an outcome precedes and causes an exposure instead of the other way around [95]. Autier et al. have performed a systematic review of vitamin D status and ill health and have concluded that the discrepancies between observational and intervention studies indicate that low 25(OH)D is a marker, not a cause, of ill health [96]. These authors have notably proposed that the wide range of disorders associated with low 25(OH)D is explained by the fact that inflammatory processes involved in disease occurrence and clinical course reduce 25(OH)D. Other groups have stressed that 25(OH)D is an unreliable biomarker of vitamin D status after an acute inflammatory insult [97].

Although subgroup analysis of a recent meta-analysis of observational studies has suggested that the inverse association of 25(OH)D level with mortality is stronger in populations with low prevalences of vitamin D supplementation or low 25(OH)D levels [76], this finding can still be attributed to reverse causation, with more severe underlying diseases being associated with lower 25(OH)D levels.

### 4.3. Mendelian Randomization

Randomization in observational studies is not possible, but an alternative exists, which is termed Mendelian randomization (MR). MR is being increasingly used to overcome confounding and reverse causation for exploring causal effects of an exposure on a disease in nonexperimental studies. The concept of MR refers to the random allocation of alleles at the time of gamete formation. By analogy with the fact that the random allocation of a treatment in a RCT renders confounding unlikely, a genetic variant of interest should not be associated with known and unknown confounding factors [98, 99]. MR studies have been conducted to infer causality for vitamin D and extraskeletal complications, such as high blood pressure and type II diabetes. Genetic variants that specifically alter 25(OH)D levels, which are usually identified from genome-wide association studies (GWAS), are generally used. For example, Kunutsor et al. have used 4 variants (in the* DHCR7*,* CYP2R1*,* GC*, and* CYP24A1* genes) as instrumental variables in a small sample (unknown *N*), failing to show a causal role of 25(OH)D in the etiology of high blood pressure [100]. Vimaleswaran et al. have used the same 4 variants but have considered allelic scores and used a large sample (*N* = 146,581), reporting that each 10% increase in genetically instrumented 25(OH)D concentration is associated with an 8% decreased odds of hypertension [101]. Using the same variants as mentioned above but not considering allelic scores, Ye et al. have also estimated the unconfounded causal associations of 25(OH)D concentration with the risks of type II diabetes and other glycemic traits using an MR approach [102], reporting insignificant MR-derived estimates for type II diabetes and glycemic traits and suggesting that the association between 25(OH)D and type 2 diabetes is not causal.

Of note, there are commonly acknowledged necessary conditions for MR to provide a causal inference in observational epidemiology [98, 99]. One condition is that the genetic instruments (e.g.,* DHCR7* variants) affect the outcome (e.g., extraskeletal complication) by no other means than through the exposure (25(OH)D), which is never the case with respect to common complex human diseases. Additionally, one cannot exclude that a true causal variant may be in linkage disequilibrium with the genetic instruments (genetic variants) used. Results obtained using the MR approach should therefore be interpreted cautiously.

### 4.4. Measurement Error and Misclassification

#### 4.4.1. Is 25(OH)D a Good Biomarker of Vitamin D Intake and/or Function?

1,25(OH)2D is the active form of vitamin D, but because 25(OH)D has a much longer circulating half-life, circulates at higher concentrations, and is less influenced by other hormones, such as PTH, it is used to determine vitamin D status. Because of its long half-life in circulation, 25(OH)D reflects vitamin D supply and usage over a period of time. Circulating 25(OH)D is also a better marker of vitamin D exposure than indirect estimates of vitamin D exposure based solely on diet, which do not take into consideration sunlight sources [103].

However, as discussed elsewhere [104], serum 25(OH)D concentration is influenced by several factors, such as the quantity of vitamin D delivered to the liver, the amount of 25(OH)D produced by the liver, and the half-life of 25(OH)D in the serum. These factors are themselves influenced by several determinants (sunlight exposure, intestinal absorption, body fat, 25-hydroxylase activity, VDBP production in the liver, etc.) [104]. Prentice et al. have stressed that because serum 25(OH)D has little interaction with VDRs, it might be a good biomarker of intake but not of function [104]. Therefore, other biomarkers of vitamin D function have been proposed (e.g., the 1,25(OH)2D/25(OH)D ratio, 1,25(OH)2D/24,25(OH)2D ratio, and >35 additional 25(OH)D metabolites formed by the body). However, evaluating such biomarkers in large epidemiological studies is difficult. Additionally, serial measurements in the same individuals are ideal but rarely feasible. Therefore, it is not excluded that 25(OH)D, when used as a biomarker of extraskeletal complications, provides biased associations similar to many other biomarkers, as demonstrated in a recent systematic evaluation of 56 meta-analyses of emerging cardiovascular biomarkers (e.g., C-reactive protein and homocysteine) [105].

Even if we agree that 25(OH)D is sufficiently linked to active 1,25(OH)D and is the* best* marker of vitamin D status similar to the conclusion of a meta-analysis performed by Tzoulaki et al. [105], it is worth noting that results vary markedly depending on the assay used [106, 107]. Liquid chromatography tandem mass spectrometry (LC-MS/MS) remains the gold standard technique to measure 25(OH)D [106, 108].

### 4.5. Deficiency* versus* Supplementation and Life-Course Perspective

Different factors that could explain the lack of replication in the few RCTs of the results reported by several observational studies have been discussed. For example, it has been suggested that the impact of vitamin D deficiency (as determined in observational studies) is not the same as the effect of vitamin D supplementation (as determined in RCTs), particularly in participants with sufficient vitamin D levels [109]. Other groups have suggested that the relationship of 25(OH)D with the risk of extraskeletal complications could be observable only at low levels of 25(OH)D [26, 109]. Davis has also suggested that the risk associated with low vitamin D status might be conferred earlier in life and that studies of circulating 25(OH)D in adults do not address the most relevant time period of exposure [26]. Vitamin D may play pivotal roles throughout life not only in calcium homeostasis and skeletal metabolism but also in infection and autoimmune disease development and progression [104]. Reviews have been published on vitamin D and functional outcomes, specifically in infants and young children [110], adolescents [111], and elderly individuals [112].

## 5. Why Results from Ongoing Randomized Controlled Trials Will Be Unlikely to Reconcile the Two Schools of Thought

**Figure 3:**
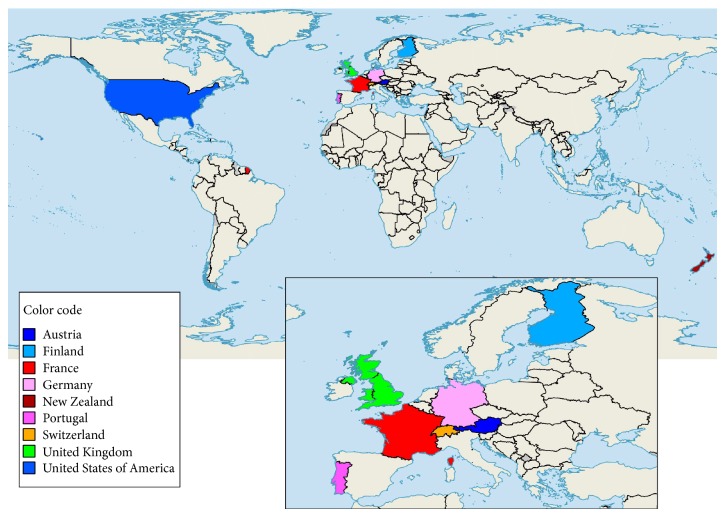
Countries where large randomized controlled trials are currently ongoing to determine the impact of vitamin D supplementation on extraskeletal complications. CAPS, USA; VITAL, USA; Do-Health, Austria, Germany, Finland, France, Portugal, Switzerland; FIND, Finland; ViDa; New Zealand. See [20] for further details.

Five RCTs (the CAPS, VITAL, Do-Health, FIND, and ViDa trials; see [20] for further details) are currently underway in 9 countries (Figure 3) to determine the impacts of vitamin D supplementation on extraskeletal complications. Most are well funded and sufficiently powered to detect a significant impact, if any exist. More than 42,000 participants will be included with a potential of 208,116 person-years, and $22 million has been invested just for the VITAL trial [113]. However, several experts have already pointed out fatal limitations that might invalidate the (negative or null) results of these trials [69, 114–117]. These limitations include the following: (i) the supplementation doses of vitamin D are too low, and higher doses should be tested; (ii) the contrast in 25(OH)D intake between the two arms is insufficient given that control groups are producing 25(OH)D even in the absence of the intervention; (iii) the 5-year follow up (which some experts have attributed to be based more on the funding grant cycle than on the vitamin D cycle) is too short; (iv) the optimal vitamin D concentration may not be the same for all extraskeletal complications considered; (v) participants are included regardless of their 25(OH)D level at baseline, and most participants already have an optimal 25(OH)D level prior to the start of the intervention. The effects of vitamin D likely depend on the baseline vitamin D status; and (vi) future trials are not likely to change pooled (null) estimates already reported by meta-analyses.

We would like to add to these limitations the potential risk of “medical reversal,” which is a phenomenon described by Prasad et al. involving the identification of faults with trials and the unwillingness to consider that an intervention might be ineffective [118]. We believe that medical reversal is even more likely to happen if ongoing trials report null effects (rather than negative effects, as in the WHI trial).

## 6. Summary and Concluding Remarks

This debate is mostly fueled by the classical divergence between observational and experimental results [119]. Observational studies are prone to reverse causation and confounding. Associations between vitamin D status and extraskeletal complications in observational studies could merely indicate that vitamin D is a “simple” indicator of health status and that compared with healthier subjects sicker subjects could have a lower vitamin D level or status. The diversity of biological systems with which vitamin D deficiency has been associated (cardiovascular, diabetes, depression, neurodegenerative diseases, cancer, etc.) could further suggest that this vitamin is a marker of health status rather than a predictor of health outcomes. However, both the wide distribution of VDRs in humans and the influence of vitamin D on more than 3% of the human genome could explain its broad effects on health.

RCTs assessing the efficacy of vitamin D supplementation in reducing extraskeletal complications are ongoing. Trials are still recruiting participants, and the first results will not be available before the year 2017. The impacts of the ongoing RCTs on clinical practice are of course difficult to predict, but at least three scenarios exist as follows: favorable and meaningful results are reported by the RCTs (scenario A); no favorable or meaningful results are reported (scenario B); or unfavorable results are reported (scenario C). All of these scenarios may or may not be followed by appropriate changes in clinical practice. Depending on the scenario, the baseline common practice, and the capacity of change in the common practice, the use of vitamin D as a biomarker could be a success or failure, as proposed by Ioannidis [120]. If it is not a success, vitamin D could eventually become a biomarker of the following: (i) “type B failure” occurs “when a biomarker shows great promise in one or more early studies, but the claims are later found to be wrong or exaggerated, and the biomarker is eventually never implemented into clinical practice” (scenario A in a practice, in which vitamin D dosing is not already common); (ii) “type D failure” occurs “when a biomarker shows no or little promise, but nevertheless is enthusiastically promoted for widespread clinical or population use” (scenario C in a practice, in which vitamin D dosing is already common); and (iii) “type A failure” occurs “when a widely used biomarker that has already been implemented in clinical practice is shown to be largely useless—or even harmful—and therefore needs to be abandoned” (scenario C in a practice, in which vitamin D dosing is already common but the common practice can be changed).

Determining 25(OH)D dosages in the absence of robust evidence seems to already be a common practice in the US, where sales of vitamin D supplements have increased from $50 million in 2005 to $600 million in 2011 [121] and where commentators already advocate clinicians to stop costly measurements of 25(OH)D in asymptomatic patients outside skeletal-related conditions [83, 122, 123].

Finally, while our review focused on extraskeletal complications and discussed the related challenges, this debate might not remain limited to extraskeletal complications for long but may soon include skeletal complications. Indeed, recent evidence has suggested that vitamin D might not be as essential as previously thought for maintaining bone health and preventing falls [64–66, 124].

Vitamin D (the solar vitamin) is likely to remain a burning topic in coming years.

## Figures and Tables

**Table 1:** Examples of different vitamin D status definitions.

Mayo clinic		Institute of Medicine (IOM)		Pilz et al.		Kidney Disease Improving Global Outcomes (KDIGO)	
PMID^*^: 20675513		PMID^*^: 21118827		PMID^*^: 21682758		PMID^*^: 19644521	
Severe deficiency	<25	At risk of deficiency	<30	Deficiency	<50	Deficiency	<37
Moderate deficiency	25–59.9	At risk of inadequate level	30–49	Insufficiency	50–74.9	Insufficiency	37–75
Optimal	60–200	Sufficient	50–125	Optimal	75–100	Adequate	>75
Possible toxicity	>200	Possible toxicity	>125	Sufficiency	75–250		
				Intoxication	>375–500		

25(OH)D expressed in nmol/L. Conversion factor for 25(OH)D: 1 ng/mL = 2.496 nmol/L; ^*^PMID: PubMed unique identification number.
